# Comparison of myopia-related behaviors among Chinese school-aged children and associations with parental awareness of myopia control: a population-based, cross-sectional study

**DOI:** 10.3389/fpubh.2025.1520977

**Published:** 2025-02-18

**Authors:** Chaoying Ye, Yujie Wang, Yujia Liu, Xingxue Zhu, Jianmin Shang, Xiaomei Qu

**Affiliations:** ^1^Department of Ophthalmology and Vision Science, Eye & ENT Hospital of Fudan University, Shanghai, China; ^2^NHC Key Laboratory of Myopia, Fudan University, Shanghai, China; ^3^Laboratory of Myopia, Chinese Academy of Medical Sciences, Shanghai, China; ^4^Department of Child and Adolescent Health, School of Public Health, Fudan University, Shanghai, China

**Keywords:** eye-use behaviors, myopia, near work time, parental awareness, school-aged children, sitting position

## Abstract

**Background:**

In China, approximately 30 and 70% of primary and middle school students, respectively, have myopia, making myopia prevention and control necessary. Eye-use behaviors are closely related to myopia, highlighting the importance of determining the behavioral compliance rates of children. Parental awareness also affects children’s behaviors. Therefore, we assessed the myopia-related behaviors and parental awareness of school-aged children in different city tiers of China with different refractive statuses.

**Methods:**

A population-based, cross-sectional study was conducted on Chinese children from 110 cities aged 7–15 years. Samples were equally allocated to each subgroup of city tiers, children’s age groups, and children’s refractive statuses. Questionnaires were designed to investigate children’s behaviors, including responses to sitting position, time of eye use (single continuous near work time and breaks in between, total near work time after school each day), eye rest (break time and style during near work and outdoor time per week), light conditions in the learning environment at home, and parents’ knowledge about myopia prevention and control. Associations between parental awareness and children’s behaviors were analyzed using logistic regression.

**Results:**

In total, 896 questionnaires were collected. The prevalence of children’s poor behaviors related to myopia ranged from 23.44 to 84.82%, with the highest and lowest being sitting position and the use of eye-protecting lamps, respectively. Children in third-tier cities were more likely to have poor sitting position (*p* < 0.01), a non-open view in front of a desk at home (*p* = 0.02), and more near activities during break times (*p* = 0.04). After adjustment for parental myopia condition and the child’s sex, poor parental awareness was mainly associated with not using an eye-protecting lamp (odds ratio [OR]: 1.95, 95% confidence interval [CI]: 1.40–2.72), poor break styles (OR: 1.60, 95% CI: 1.21–2.12), and excessive total near work time (OR: 1.45, 95% CI: 1.02–2.05).

**Conclusion:**

Myopia-related behaviors were poorly performed in children, particularly among older children and those living in third-tier cities. Eye-protecting lamps, time spent doing near work, and break style were all associated with parental awareness, suggesting that better parental awareness helps children in the long run. More targeted measures could be adopted to help improve children’s behaviors.

## 1 Introduction

Myopia has become a major public health concern globally due to its rising prevalence, and approximately 30% of the global population is currently myopic (1), with a tendency toward earlier onset, increased severity, and a higher incidence of high myopia. It is estimated that by 2050, nearly 50% of the world’s population will be myopic, and 10% will have high myopia (2), which will inevitably result in far more severe vision impairment, such as retinal detachment, cataracts, glaucoma, and myopic macular degeneration (3, 4), and pose a heavy burden on individuals and society (5).

Currently, the prevalence of myopia in children is a major public health concern, especially in East Asian countries and regions. The first publication on eye health in China, titled “White Paper on Eye Health in China,” was released by the National Health Commission in 2020. According to the results presented, the nationwide incidence of myopia in children and teenagers was 53.6%. Among the demographic groups under consideration, the prevalence of myopia was 14.5% among 6-year-olds, 36.0% among primary school students, and 71.6% among middle school students. Furthermore, it was observed that the prevalence of myopia was highest among senior high school students, with a rate of 81.0%, which is significantly higher than that in other countries such as Germany (6) and Nepal (7). The prevalence of myopia in other Asian countries (such as Japan, South Korea, Singapore) also remains high, with myopia rates reaching 50–70% among school-age children. In Europe and the United States, the prevalence of myopia is relatively low, for example, about 30–40% in the United States, but in recent years, due to factors such as the increased use of digital screens, myopia prevalence among teenagers is also on the rise (8, 9). Furthermore, myopia can affect a child’s education and quality of life. In addition to decreasing visual comfort, uncorrected refractive errors can easily impact a child’s life experiences and participation in the classroom. In severe cases, myopia can result in irreversible amblyopia, which can cause psychological distress, social isolation, and limited opportunities for future education and employment (10–12). Given the urgent situation of myopia and the fact that its etiology remains unclear, strategies to reduce its prevalence and development in China are increasingly warranted.

Although hereditary and environmental factors are the primary causes of myopia, it is presently thought that genetic changes play a minor role and environmental factors have a more significant impact (13). Environmental factors include eye-use behaviors such as outdoor activities, near work time, and the use of digital devices. Myopia in teens can be prevented and delayed by developing good habits in daily life (14–16). Many studies have indicated that more time spent outside can reduce the incidence of myopia (17–19). Aslan et al. (20) found that those who spent 2 h a day outside were 33% less likely to have worsened myopia. He et al. (21) conducted a study that showed that adding 40 min of outdoor activity at school compared with the usual activity resulted in a reduced incidence rate of myopia over 3 years among 6-year-old children in Guangzhou, China. Studies have also shown that single continuous periods of eye use have a greater effect on myopia than does the total time of near-eye use and that myopia can be prevented by giving the eyes enough time to rest (22). Furthermore, parental awareness may directly impact children’s behaviors and serve a supervisory function. Studies have revealed that parents can prevent their children from becoming overweight by modifying their diets and encouraging them to be more physically active (23, 24); therefore, their attitudes can influence children’s physical activities and screen time (25). However, few studies have focused on parental awareness and the impact of home environment formation on children’s behaviors. Furthermore, most cross-sectional studies are geographically limited, failing to present an analysis of the nationwide population (26–30). The Chinese government has issued guidelines for myopia prevention and control; however, the implementation of these guidelines across regions with different levels of economic development has not yet been reported.

Therefore, in this study, we investigated differences in children’s behaviors and parental awareness of myopia from the perspectives of different cities, age groups, and refractive status and further explored the influence of awareness on behaviors to describe the current state of myopia prevention and control in China and hope to contribute to more targeted policy implementation in the future.

## 2 Materials and methods

### 2.1 Study participants

This population-based, cross-sectional study was conducted in China from January to April 2022. The study was approved by the Ethics Committee of the Eye & ENT Hospital of Fudan University, and all procedures were conducted in compliance with the tenets of the Declaration of Helsinki.

We obtained information about the children’s behaviors and parental awareness through questionnaires completed by parents. To obtain a representative sample, a stratified sampling approach was used to recruit respondents stratified based on city tier (1-, 2-, and 3-tier cities), children’s age group (7–9, 10–12, and 13–15 years), and refractive status (no myopia, mild and moderate myopia, and high myopia). The division of Chinese cities is mostly determined by the region’s economic strength, city size, regional radiation power, and population size. According to the documents released by the Chinese National Bureau of Statistics (31), first-tier cities, including Beijing, Shanghai, Guangzhou, Shenzhen, are often economically developed and have abundant educational facilities. Second-tier cities include most provincial capitals such as Hangzhou and Nanjing, while third-tier cities are small and medium-sized cities such as Luoyang, reflecting to a certain extent the ranking of economic, cultural and educational levels from high to low. Children aged 7–15 years were selected as the target age as they are school-age children in China and are also at higher risk of myopia onset, faster progression, and higher potential impact on the future (8). Divided into 7–9 as early elementary school, 10–12 as late elementary school, and 13–15 as middle school, respectively, to gain specific understanding. Refractive status was classified as follows (32, 33): mild and moderate myopia (low myopia) was defined as the eye with a spherical equivalent (SE) refractive error of ≤ − 0.50D and > −6.00D, while high myopia was defined as ≤ − 6.00D. No myopia was defined as spherical equivalent > − 0.50D. The sample size was determined by assuming 50% of the prevalence of myopia-related behaviors, with a confidence level of 95%, a relative error of 10%, a design effect of 2, and a nonresponse rate of 10%. The estimated minimum sample size was 845, allocated equally to each subgroup.

The respondents provided relevant information by completing questionnaires distributed online through the Tencent platform.^1^ Questionnaire distribution was stopped once the number of eligible questionnaires reached the planned quantity, and completed questionnaires were excluded if (1) the child was younger than 7 years old or older than 15 years old, (2) the child had an eye disorder or a systemic disease, other than myopia, and (3) the parent was engaged in industry or market research related to eye care. Subsequently, the questionnaires were manually screened for those lacking plausibility, such as those with contradictory answers to previous and subsequent questions.

### 2.2 Questionnaire

The questionnaires mainly covered children’s information and parents’ awareness of myopia control. The children’s information encompassed demographics (age, sex, region, and medical history), their own and parental refractive status, and their parents’ occupations. Medical history is investigated to exclude ocular conditions that could interfere with the evaluation, primarily active eye diseases such as cataracts, glaucoma, or conical cornea, as well as changes in the eyes brought on by systemic disorders such as autoimmune diseases (like systemic lupus erythematosus, rheumatoid arthritis), genetic diseases (like Marfan syndrome), and metabolic diseases (like hypertension, diabetes, hyperthyroidism, etc.). The refractive status was obtained by self-completion in the questionnaire. Children’s behaviors were investigated, including their sitting position, light conditions in the learning environment at home [view in front of the desk at home, lighting in the learning environment, and the use of eye-protecting lamps, which refers to the series of desk lamps whose performance meets the Chinese national regulations (34)], time of eye use (single continuous near work time and breaks in between, total near work time after school each day), and eye rest (break time and style during near work and outdoor time per week). Parents’ awareness of myopia included the following: awareness of the prevention and control of myopia and high myopia; timing of parental awareness (the time when parents became aware that myopia can be prevented and controlled before or after their child’s diagnosis); knowledge of the harms of myopia, pathological myopia, and myopia correction; motivation to bring their children for regular comprehensive eye examinations; and habit of keeping the reports.

The aforementioned issues regarding awareness were equally weighted, with a total score of 9. The scores of each item were uniformly converted into 0, 0.5, and 1, corresponding to “do not know” or “wrong answer, right answer but unfamiliar with the knowledge,” and “right answer and familiar with the knowledge,” respectively.

Parents with a score greater than or equal to *P*50 (5 points) were defined as having good awareness of myopia.

### 2.3 Classification of behaviors

The original categories of behaviors were dichotomized according to the advice provided in the Chinese guidelines (35). Proper sitting position was defined as maintaing the “one fist, one foot, one inch”rule without reminder; otherwise, it was considered poor. For study environments at home, two types of desk views were identified: an “open view,” where the desk was fixed near a window with good lighting, and a “non-open view,” where this condition was not met. Good lighting was defined as the combined use of desk lamps and ambient light, while bad lighting involved using only one of these. Desk lamps were further classified as general table lamps and eye-protecting lamps, with the latter being preferred.

Single continuous near work time was defined as the duration of a single continuous near-eye use, and break time was defined as the interval between two continuous near work activities. Additionally, total near work time means the amount of time spent at a near distance to activities after school each day, including reading, doing homework, using a phone or tablet, attending hobby classes, and tuitions. Outdoor time was defined as the time spent outside per week. Children were considered to have beneficial behaviors if they had <40 min of single continuous near work time, more than 10 min of break time, more than 14 h of outdoor time per week, and <2 h of total near work time per day.

A beneficial break style during near work was defined as no near activities performed during the break time. No near activities included doing outdoor activities, looking away through a window, using eye-relaxing methods such as massagers, steaming eye masks, or resting with eyes closed.

### 2.4 Statistical analysis

We reorganized the samples into two sections, children’s behavior and parental awareness, and used SPSS v26.0 (SPSS Inc., Chicago, IL, USA) to analyze the data. Categorical variables are expressed as frequencies with percentages and compared among groups using the chi-squared test. Metric variables were presented as medians and quartiles. Multivariate logistic regression analysis determined the association between region, age groups, refractive status, and children’s behavior. *p* < 0.05 were considered statistically significant in this study.

## 3 Results

### 3.1 Participant characteristics

Table 1 presents the relevant demographic characteristics of the respondents. We enrolled 896 parents of children aged 7–15 with a qualified questionnaire response rate of 99.56%. They came from four first-tier cities (Shanghai, Beijing, Guangzhou, and Shenzhen), 22 s-tier cities, and 84 third-tier or lower cities across China.

**Table 1:** Demographic characteristics of children and parents.

Characteristics	Number of respondents (*N* = 896)	Proportion (%)
Refractive status of children		
No myopia	300	33.48
Mild and moderate myopia	300	33.48
High myopia	296	33.04
Age of children (years)		
7–9	300	33.48
10–12	298	33.26
13–15	298	33.26
City level		
First-tier cities	279	31.14
Second-tier cities	307	34.26
Third-tier or lower cities	310	34.60
Child’s sex		
Boy	460	51.34
Girl	436	48.66
Parent’s sex		
Male	279	31.14
Female	617	68.86
Parental myopia		
Neither	263	29.35
Either	349	38.95
Both	284	31.70

### 3.2 Distribution of children’s behaviors

**Figure 1:**
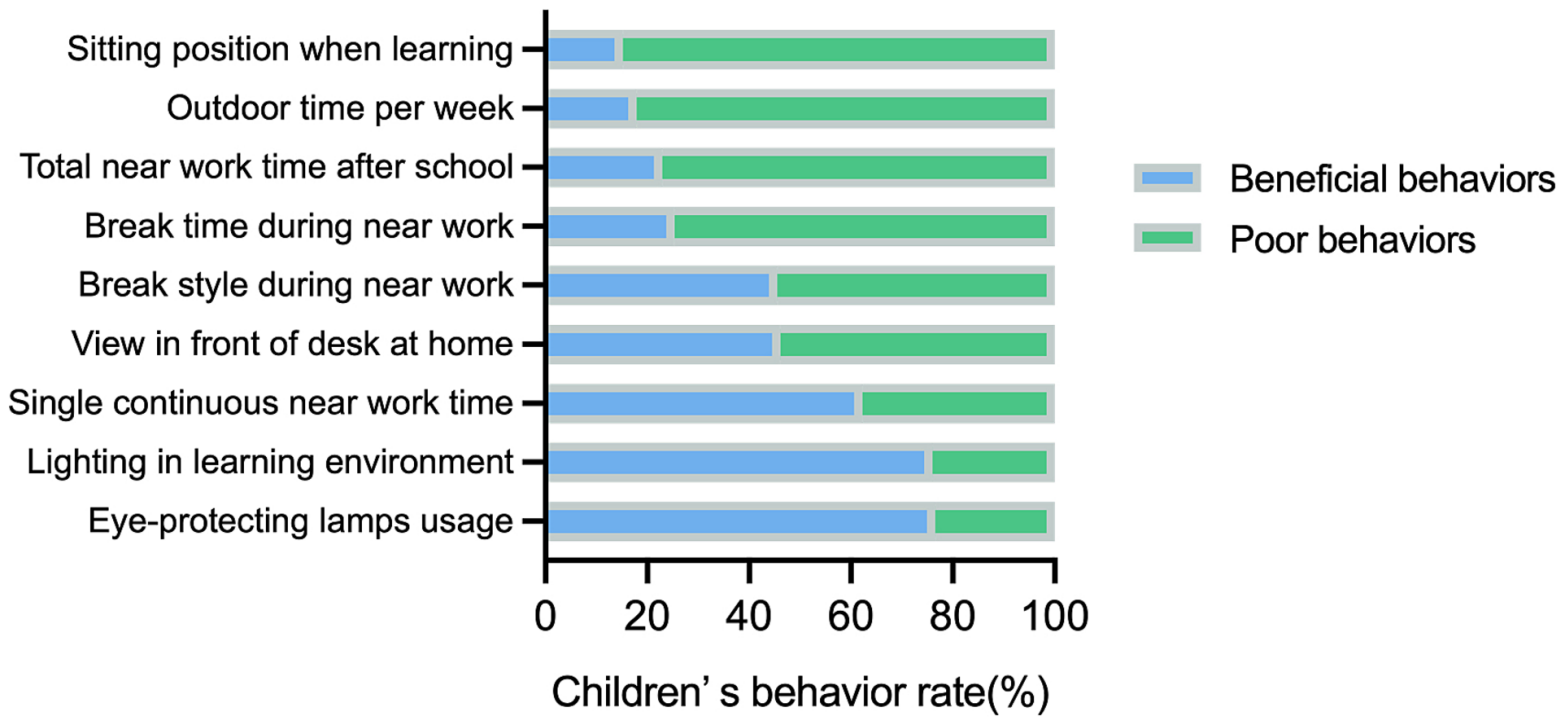
The total rate of various eye-related behaviors in children across city tiers, refractive status, and age groups.

Overall, the children exhibited a high proportion of poor behaviors during eye use and eye rest (Figure 1 and Table 2). The rate of sitting position was the highest among all investigated behaviors, reaching 84.82%, followed by outdoor time, with a percentage of insufficient outdoor time of 82.17%. However, the highest proportion of good behavior was found in creating learning environments with good lighting at home and using eye-protecting lamps, reaching 76.00 and 76.56%, respectively.

**Table 2:** Comparisons of children’s eye-related behaviors across city tiers, refractive status, and age groups.

Behaviors	Total rate	City tier			Refractive status of children			Age group of children (years)		
	*N* (%)	First-tier (*N* = 279)	Second-tier (*N* = 307)	Third-tier (*N* = 310)	No myopia (*N* = 300)	Mild and moderate myopia (*N* = 300)	High myopia (*N* = 296)	7–9 (*N* = 300)	10–12 (*N* = 298)	13–15 (*N* = 298)
Sitting position										
Good	136 (15.18)	57 (20.43)	44 (14.33)	35 (11.29)	95 (31.67)	31 (10.33)	10 (3.38)	37 (12.33)	41 (13.76)	58 (19.46)
Poor	760 (84.82)	222 (79.57)	263 (85.67)	275 (88.71)	205 (68.33)	269 (89.67)	286 (96.62)	263 (87.67)	257 (86.24)	240 (80.54)
*P*		<0.01*			<0.01*			0.04*		
View in front of the desk at home										
Open	413 (46.09)	137 (49.10)	153 (49.84)	123 (39.68)	136 (45.33)	149 (49.67)	128 (43.24)	134 (44.67)	132 (44.30)	147 (49.33)
Not open	483 (53.91)	142 (50.90)	154 (50.16)	187 (60.32)	164 (54.67)	151 (50.33)	168 (56.76)	166 (55.33)	166 (55.70)	151 (50.67)
*P*		0.02*			0.275			0.39		
Lighting in the learning environment										
Good	681 (76.00)	220 (78.85)	223 (72.64)	238 (76.77)	215 (71.67)	215 (71.67)	251 (84.80)	228 (76.00)	233 (78.19)	220 (73.83)
Bad	215 (24.00)	59 (21.15)	84 (27.36)	72 (23.23)	85 (28.33)	85 (28.33)	45 (15.20)	72 (24.00)	65 (21.81)	78 (26.17)
*P*		0.20			<0.01*			0.46		
Use of an eye protection lamp										
Yes	686 (76.56)	213 (76.34)	232 (75.57)	241 (77.74)	213 (71.00)	249 (83.00)	224 (75.68)	239 (79.67)	221 (74.16)	226 (75.84)
No	210 (23.44)	66 (23.66)	75 (24.43)	69 (22.26)	87 (29.00)	51 (17.00)	72 (24.32)	61 (20.33)	77 (25.84)	72 (24.16)
*P*		0.81			<0.01*			0.27		
Single continuous near work time (minutes)										
<=40	558 (62.28)	184 (65.95)	189 (61.56)	185 (59.68)	196 (65.33)	176 (58.67)	186 (62.84)	219 (73.00)	182 (61.07)	157 (52.68)
>40	338 (37.72)	95 (35.05)	118 (38.44)	125 (40.32)	104 (34.67)	124 (41.33)	110 (37.16)	81 (27.00)	116 (38.93)	141 (47.32)
*P*		0.28			0.24			<0.01*		
Break time during near work (minutes)										
>10	227 (25.33)	66 (23.66)	87 (28.34)	74 (23.87)	65 (21.67)	78 (26.00)	28 (28.38)	83 (27.67)	94 (31.54)	50 (16.78)
≤10	669 (74.67)	213 (76.34)	220 (71.66)	236 (76.13)	235 (78.33)	222 (74.00)	212 (71.62)	217 (72.33)	204 (68.46)	248 (83.22)
*P*		0.33			0.16			<0.01*		
Break style during near work										
No near activities	408 (45.54)	136 (48.75)	149 (48.53)	123 (39.68)	149 (49.67)	122 (40.67)	137 (46.28)	127 (42.33)	143 (47.99)	138 (46.31)
Including near activities	488 (54.46)	143 (51.25)	158 (51.47)	187 (60.32)	151 (50.33)	178 (59.33)	159 (53.72)	173 (57.67)	155 (52.01)	160 (53.69)
*P*		0.04*			0.08			0.36		
Total near work time (hours per day)										
≤2	205 (22.88)	74 (26.52)	68 (22.15)	63 (20.32)	78 (26.00)	68 (22.67)	59 (19.93)	111 (37.00)	59 (19.80)	35 (11.74)
>2	691 (77.12)	205 (73.48)	239 (77.85)	247 (79.68)	222 (74.00)	232 (77.33)	237 (80.07)	189 (63.00)	239 (80.20)	263 (88.26)
*P*		0.19			0.21			<0.01*		
Outdoor time (hours per week)										
≥14	160 (17.86)	59 (21.15)	51 (16.61)	50 (16.13)	53 (17.67)	50 (16.67)	57 (19.26)	99 (33.00)	45 (15.10)	16 (5.37)
<14	736 (82.14)	220 (78.85)	256 (83.39)	260 (83.87)	247 (82.33)	250 (83.33)	239 (80.74)	201 (67.00)	253 (84.90)	282 (94.63)
*P*		0.22			0.71			<0.01*		

^*^P < 0.05; data were analyzed using the chi-squared test. N, number.

The myopia-related behaviors of the children were compared using the chi-squared test (Table 2). Children in third-tier cities were likelier to have poor sitting position, a non-open view in front of a desk at home, and to engage in near work activities during break times. Moreover, those with myopia had a higher proportion of learning environments with good lighting at home than did those without myopia. Refractive status and city tiers were not associated with near work, break time, or outdoor time, whereas these behaviors worsened with increasing age. The proportion of longer single continuous near work time and total near work time increased, while the proportion of break time and weekly outdoor time meeting the requirements declined. The percentage of good sitting position grew, although it did not exceed the maximum of 20%.

### 3.3 Parental awareness of myopia control

Table 3 shows the parents’ scores on the responses to myopia-related questions and the assessed good awareness rates. The total score was 9, with a median score of 5, for all data used to define good or poor awareness.

**Table 3:** Differences in parental awareness among city tiers, refractive status, and age groups.

Children’s characteristics	Scores of parental awareness M (IQR)	Rates of good awareness *N* (%)	*P*
City level			
First-tier	5.5 (4.0, 6.5)	176 (63.08)	
Second-tier	5.0 (3.0, 6.0)	165 (53.75)	<0.01
Third-tier	4.5 (3.0, 5.6)	147 (47.42)	
Refractive status			
No myopia	4.5 (3.0, 6.0)	132 (44.00)	
Mild and moderate myopia	5.5 (4.0, 7.0)	196 (65.33)	<0.01
High myopia	5.0 (3.5, 6.0)	160 (54.05)	
Age group (years)			
7–9	5.5 (4.0, 6.5)	187 (62.33)	
10–12	5.0 (3.5, 6.0)	172 (57.72)	<0.01
13–15	4.5 (2.5, 6.0)	129 (43.29)	
Overall	5.0 (3.5, 6.0)	488 (54.46)	

Rates of awareness were compared using a chi-squared test. M, mean; IQR, interquartile range; N, number.

The average score for awareness of myopia control was 5.0, and 54.5% of parents had relatively good awareness. Parents who lived in more developed cities had younger children with myopia and were found to have a better awareness of myopia control (*p* < 0.01).

### 3.4 Factors associated with children’s behaviors

**Figure 2:**
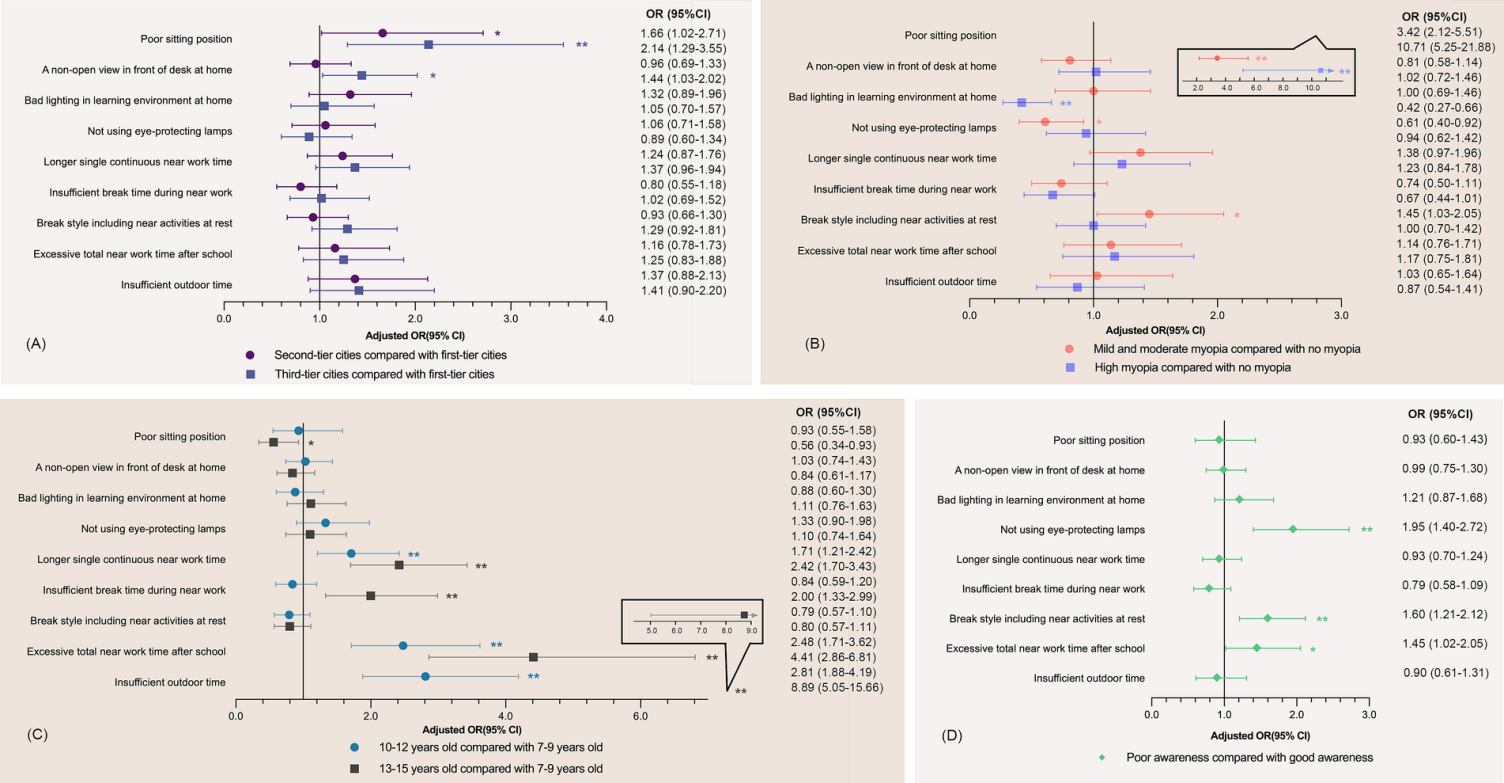
Association between myopia-related behaviors and city tiers, refractive status, age groups, and parental awareness of myopia control. *p < 0.05; **p < 0.01; OR, odds ratio; CI, confidence interval. Data were analyzed using multivariate logistic regression analysis. Adjusted for parental myopia (neither vs. either vs. both) and sex (male vs. female). Longer continuous near work time: near-eye use of more than 40 min each time; insufficient break time during near work: break time less than 10 min each time; break style including near activities at rest: doing both no near activities and near activities during break time; excessive total near work time after school: total near work time of more than 2 h after school each day; insufficient outdoor time: outdoor time of fewer than 14 h per week. **(A)** Association between city tiers and children’s behavior; **(B)** Association between the refractive status of children and children’s behavior; **(C)** Association between the age groups of children and children’s behavior; **(D)** Association between parental awareness of myopia control and children’s behavior. A good sitting position, an open view, good lighting conditions, the use of eye-protecting lamps, continuous near work time of fewer than 40 min, a break time of more than 10 min, no near activities during breaks, near work time of fewer than 2 h, and weekly outdoor time of more than 14 h were selected for the control groups.

The results of the multivariate logistic regression analysis of the association between children’s behaviors and city tiers, refractive statuses, age groups, and parental awareness are shown in Figure 2, adjusted for parental myopia and the child’s sex.

The different city tiers were mainly associated with sitting position and views from the desk. Compared with children in first-tier cities, those in second- and third-tier cities had 0.66 (95% confidence interval [CI]: 1.02–2.79, *p* < 0.05) and 1.14 (95% CI, 1.29–3.55, *p* < 0.01) times more possibility of having poor sitting position. Compared with the non-myopic population, children with myopia had a poorer sitting position but paid more attention to creating good lighting conditions in the learning environment at home. Older children tended to have good sitting position but poor performance in eye use and eye rest, like longer single continuous near work time, excessive total near work time after school, insufficient break time and outdoor time.

Poor parental awareness was associated with excessive total near work time outside school (odds ratio [OR]: 1.45, 95% CI: 1.02–2.05, *p* < 0.05), no use of eye-protecting lamps (OR: 1.95, 95% CI: 1.40–2.72, *p* < 0.01), and doing more near activities at rest (OR: 1.60, 95% CI: 1.21–2.12, *p* < 0.01), but not with time outdoors (OR: 0.90, 95% CI: 0.61–1.31, *p* > 0.05).

## 4 Discussion

In this study, we analyzed children’s myopia-related behaviors, parental awareness across city tiers, age groups, and refractive statuses, and factors associated with these behaviors. The sitting position varied significantly across the comparison categories. Children in third-tier cities with a less open view at their desks and children with myopia were more likely to have beneficial lighting conditions in the learning environment at home, and those in older age groups performed worse in terms of eye use and rest. Additionally, parental awareness of myopia control varied significantly by categorical factors and was associated with eye-protecting lamps, total near work time, and break style.

The behaviors of children showed some differences across cities, notably in their sitting position, averaging 15.18%. First-tier cities, non-myopia groups, and older age groups had higher adherence rates to good sitting position. The total rate was higher than that reported by Wang et al. (36) in Wenzhou; however, the rate was the same in children with high myopia. Differences in survey methodology and geographical coverage may explain the variation in reported rates. Meanwhile, those in third-tir city also had higher proportion in having a “not-open view” while studying. This could be because that first- and second-tier cities have more access to medical and educational facilities, so that teachers and parents would pay more attention to myopia education and, as a result, observing and reinforcing children’s behavioral tendencies. In addition, with age, children’s awareness and attention improve, resulting in better sitting position. Moreover, a consistently poor sitting position poses a risk factor for the development and progression of myopia. According to Wang et al. (36), it could amplify the risk of common myopia and high myopia by 7.2 and 9.0 times, respectively. Pärssinen et al. (37) discovered that various gaze directions induce distinct tensions in the extraocular muscles, impacting the eyeballs’ pressure and leading to eye axis lengthening (38). Therefore, children with high levels of myopia often exhibit poor sitting position. Given the challenging situation, advocating for adjustable desks and corrective posture equipment among children in the lower grades could be beneficial. The Guidelines on Appropriate Technology for the Prevention and Control of Myopia in Children and Adolescents advises kids to maintain proper reading and writing posture, so that “one fist, one foot, one inch”; not to lie down to read, and not to read or use electronic devices while walking, eating, etc. Furthermore, additional education is necessary to help them avoid poor habits while doing near work activities (39).

There was a high level of eye overuse among the respondents in this study. Of the children, 77.12% spent more than 2 h a day on near work, especially older children. Working at a distance of <20 cm for up to 2 h per day is a risk factor for myopia (40, 41). Fu et al. (42) confirmed that a short viewing distance may be a risk factor for myopia in guinea pig models. This may be because accommodation lag increases with decreasing reading distance, which induces hyperopic retinal defocus and myopia progression (43). The relevant provisions also state that written homework should not exceed 60 min in grades 3–6, while it should not exceed 90 min in middle school, and electronic devices should not be used for more than 15 min in a single session and not more than 1 h cumulatively per day (39).

In our study, children spent less time outdoors per week with increasing age, with a compliance rate of only 17.86%, whereas many studies have indicated that spending more time outdoors during childhood is associated with a reduced risk of myopia (17, 19, 20, 44). Children are recommended to take a minimum 10-min break after every 40 min of near work (39); however, only 25.33 and 62.28% adhered to these requirements, indicating that the current situation regarding children’s eye rest in China falls short of the recommended guidelines. Some studies have suggested that myopia may be prevented and delayed by getting enough rest for the eyes (14, 19, 45) and is more likely to be accelerated by a single extended period of eye use as opposed to the total time spent on near work (22, 46).

These poor behavioral performances can be attributed to the increased academic demands prevalent in educational systems. Reduced outdoor time is often observed with extensive education-related near work (47), with recess and sports classes commonly taken up by indoor teaching activities, which worsen progressively with age. In addition, the lack of an outdoor atmosphere in the home or neighborhood can make children less motivated to participate actively.

We also found an association between parental awareness and near work time and break style but not outdoor time. This is consistent with a study in Taiwan (28), potentially indicating that parental awareness is still lagging behind clinical recommendations. Notions such as discouraging long periods of single continuous near work and encouraging outdoor time are not sufficiently widespread. On the other hand, parental influence over their children may not be sufficient to help children resist increasing academic pressure, which also significantly impacts their behaviors. Consequently, schools could pay more attention to reducing the academic burden, reducing the occupation of students’ recess hours, and providing eye care education, especially for older students. Parents should increase their awareness and take the initiative to encourage their children to spend time outdoors (48), prompt them to take breaks by looking away and help them engage in eye exercises to relax between periods of continuous reading. Outpatient doctors also play an active role in education.

Considering that the use of standard LED lamps and dim reading environments was not conducive to the prevention and control of myopia (49, 50), the observation that children with myopia in this study had better lighting and a higher likelihood of using eye-protecting lamps in their home learning environments could be linked to concerns about exacerbating myopia (48, 49). However, more than 30% of the children with low myopia and children without myopia still had poor lighting conditions, and less than half of the respondents had an open view from their desks, with the majority facing the wall. Priority should be given to establishing conducive learning environments for children with non-high myopia to help maintain the myopia at a low level or prevent its onset. Furthermore, good parental awareness promoted eye-protecting lamps, probably due to the ease of obtaining and utilizing them. It required less time, effort, and resistance than did counter educational pressures and external environmental factors, facilitating their implementation. Better dissemination of relevant education is needed to create a more conducive home environment for learning. Furthermore, schools can pass on the knowledge of myopia prevention and control to parents, establish vision examination files, and promptly notify parents of the results of school vision checkups, so as to prevent and control myopia early in their children’s lives. Some studies have found that by sending regular messages to parents was able to increase the amount of time spent outdoors in children over the age of three, slowing the progression of myopia and reducing the prevalence of myopia (51–53). Schools can also host lectures on the topic to address their questions in person.

This study identified participation by city, age, and refractive status ratio to exclude interference when examining a single factor. Rather than a hospital-based study, we conducted a population-based study to examine children’s behaviors and parental awareness, providing a complete picture of myopia prevention and control. The sample size was sufficiently large to be representative. However, the study has some limitations. First, the information, including behaviors and awareness, was gathered from questionnaires; thus, recall bias may exist and may have decreased the reliability. Second, it was a cross-sectional study with no identified causal association. Therefore, multicenter cohort studies could further demonstrate the relationship between parental awareness of myopia control, children’s behavior, and myopia risk.

In conclusion, by comparing the behaviors of children with different characteristics across China, we found that myopia-related behaviors were poorly performed in children, particularly among older children and those with myopia. In addition, eye-protecting lamps, time spent near work, and break style were all associated with parental awareness. Multiple strategies, including parental guidance, children’s awareness development, and school-based education programs, are necessary to cultivate children’s myopia-related behaviors. Moreover, more studies are needed to understand the causal relationship between awareness and behaviors and assess children’s behavior improvements. This study can help initiate and augment resource planning and infrastructure development for preventing and correcting myopia, especially in rural schoolchildren, to tackle this issue before it becomes an epidemic.

## Data Availability

The raw data supporting the conclusions of this article will be made available by the authors, without undue reservation.

## Footnotes

^1^ https://wj.qq.com/index.html
