# Supplementary material for: Comparison of myopia-related behaviors among Chinese school-aged children and associations with parental awareness of myopia control: a population-based, cross-sectional study
Source: Front Public Health. 2025 Feb 18;13:1520977. doi: 10.3389/fpubh.2025.1520977 (PMC11876055; doi:10.3389/fpubh.2025.1520977)
Supplement: Supplementary file 1 [file Data_Sheet_1.docx]

Part I

Q1_1【Single choice】Child's Age

Options for this question:

1.1

2.2

3.3

4.4

5.5

6.6

7.7

8.8

9.9

10.10

11.11

12.12

13.13

14.14

15.14

Q1_2【Selection】In which province and city is your child located?

Options for this question:

1.Please select a province

2.Please select a city

3.Please select a district/county

Q1_3.【Single choice】What is your child's gender?

Options for this question:

1.Male

2.Female

Q1_4.【Single choice】What grade is your child in?

Options for this question:

1.Not yet in kindergarten

2.Kindergarten - Junior Class

3.Kindergarten - Middle Class

4.Kindergarten - Senior Class

5.Primary School - Grade 1

6.Primary School - Grade 2

7.Primary School - Grade 3

8.Primary School - Grade 4

9.Primary School - Grade 5

10.Primary School - Grade 6

11.Junior High School - Grade 1

12.Junior High School - Grade 2

13.Junior High School - Grade 3

14.Senior High School - Grade 1

15.Senior High School - Grade 2

16.Senior High School - Grade 3

Q1_5. 【Single choice】Is your child myopia now?

Options for this question:

1.Yes

2.No

Q1_5_1. 【Fill in the blank】What was the degree of your child's last vision screening?

Left eye: ___ Right eye: ___

Q1_6. 【Multiple choice】Does your child have any of the following eye diseases?

1.Fundus diseases (such as macular diseases, fundus vascular diseases, retinal detachment, congenital fundus abnormalities)

2.Ocular surface diseases (such as keratoconus)

3.Cataracts

4.Amblyopia

5.Other congenital eye diseases

6.Color blindness

7.None of the above eye diseases

Q1_7. 【Multiple choice】Does your child have any of the following systemic diseases?

1.Autoimmune diseases (such as systemic lupus erythematosus, rheumatoid arthritis, etc.)

2.Metabolic diseases (such as diabetes, hyperthyroidism, etc.)

3.Genetic diseases (such as Marfan syndrome, etc.)

4.Anemia

5.Other systemic diseases

None of the above systemic diseases

Part II

Q2_1.【Single choice】You are the child's _____?

Options for this question:

1.Father

2.Mother

Q2_2.【Single choice】What is the child's father's occupation?

Options for this question:

1.Optometry-related work (such as eyewear industry practitioners, optometrists, optometry education)

2.Market research work

3.Ophthalmology-related work (such as ophthalmologists, nurses, medical students, etc.)

4.Not the above occupations

Q2_3.【Single choice】What is the child's mother's occupation?

Options for this question:

1.Optometry-related work (such as eyewear industry practitioners, optometrists, optometry education)

2.Market research work

3.Ophthalmology-related work (such as ophthalmologists, nurses, medical students)

4.Not the above occupations

Q2_4.【Single choice】Are you myopia? If so, what is the degree?

Options for this question:

1.myopia

2.No myopia

Q2_5. What is the degree?

Options for this question:

1.<600 degrees

2.≥600 degrees

Q2_6.【Multiple choice】What other eye diseases do you have?

Options for this question:

1.Glaucoma

2.Cataracts

3.Nystagmus

4.Hereditary retinal degeneration

5.Others

6.None of the above eye diseases

Q2_7.【Single choice】Is your spouse nearsighted? If so, what is the degree?

Options for this question:

1.myopia

2.No myopia

Q2_8. What is the degree?

Options for this question:

1.<600 degrees

2.≥600 degrees

Q2_9.【Multiple choice】What other eye diseases does your spouse have?

Options for this question:

1.Glaucoma

2.Cataracts

3.Nystagmus

4.Hereditary retinal degeneration

5.Others

6.None of the above eye diseases

Part III

Q3_1.【Single choice】What is your child's usual sitting posture when doing close-up eye work?

Options for this question:

1.Consciously maintain a proper sitting posture, that is, meet the requirements of "one fist, one foot, one inch", and can maintain it all the time when doing close-up eye work

2.The child's initial sitting posture is proper, but later needs parental supervision

3.The child's sitting posture is not proper, and only becomes proper with parental supervision and reminders

4.The child's sitting posture is not proper, and does not change even with parental supervision and reminders

Q3_2.【Single choice】What is directly in front of your child's desk?

Options for this question:

1.A window with good lighting

2.A window, but the lighting is average or poor

3.A wall

There is no desk for the time being

Q3_3.【Single choice】Under which of the following circumstances does your child usually use a desk lamp?

Options for this question:

1.When turning on the desk lamp, only the desk lamp is on in the room, and other places are dark

2.When turning on the desk lamp, other lights in the room are also on, and other places are also bright

Q3_4.【Single choice】Do you arrange light sources at home to protect your child's eyes, such as using eye-protecting lamps (Conforms to the standard GB 40070-2021)?

Options for this question:

1.Yes

2.No

3.Others

Q3_5_1.【Single choice】When your child does close-up eye work for a long time, how often do they usually take a break?

Options for this question:

1.<=40 minutes

2.40 minutes

3.No break

Q3_5_2.【Single choice】How long does your child usually take a break each time?

Options for this question:

1.<=10 minutes

2.10 minutes

Q3_5_3.【Multiple choice】What does your child usually do during the break?

Options for this question:

1.Go to the bathroom

2.Eat/drink

3.Close eyes to rest

4.Apply warm compress to eyes

5.Wear a steam eye mask

6.Do eye exercises

7.Use an eye massager

8.Participate in outdoor activities during the day

9.Look into the distance (look out of the window)

10.Watch TV/movies

11.Play with mobile phone/tablet

12.Read e-books

13.Read paper books

14.Play games

15.Play with small toys (such as puzzles, etc.)

16.Others

Q3_6.【Single choice】How long does your child spend on close-up eye work after school every day (including completing homework, using electronic products, attending interest classes, extracurricular tutoring and other activities)?

Options for this question:

1.<=2 hours

2.2 hours

Q3_7_1. 【Single choice/Fill in】Does your child usually do outdoor activities during the day? (Please first check whether to participate in outdoor activities during the day and fill in the corresponding total weekly duration)

Options for this question:

1.Yes

2.No

Q3_7_2. Fill in the corresponding total weekly duration Weekly ___ hours (Monday to Sunday)

Part IV

Q4_1_1. 【Single choice】Do you think myopia in children can be prevented?

Options for this question:

1.Yes

2.No

3.Not sure

Q4_1_2. 【Single choice】When did you realize that myopia in children can be prevented?

Options for this question:

1.Before the child was diagnosed with myopia

2.After the child was diagnosed with myopia, informed by the doctor

Q4_2. 【Single choice】Do you think that after a child becomes nearsighted, the deepening of the myopia degree can be controlled?

Options for this question:

1.Yes

2.No

3.Not sure

Q4_3. 【Single choice】Do you think that after a child becomes nearsighted, the progression to high myopia can be controlled?

Options for this question:

1.Yes

2.No

3.Not sure

Q4_4.【Single choice】Which degree of myopia do you think is more likely to cause pathological or structural changes in the eyes?

Options for this question:

1.Mild, moderate, and high myopia

2.Moderate and high myopia

3.Only high myopia

4.Not sure

Q4_5_1. Have you ever heard of pathological myopia?

Options for this question:

1.Never heard of it

2.Heard of it but not fully understood

3.Heard of it and fully understood

Q4_5_2【Multiple choice】What is your understanding of pathological high myopia?

Options for this question:

1.Pathological myopia will progress throughout life

2.The axial length of the eye will excessively increase in pathological myopia

3.Pathological myopia is prone to fundus changes

4.Pathological myopia will not lead to irreversible visual damage

Q4_6. Which of the following statements about myopia correction surgery (such as laser surgery, lens implantation, etc.) is correct?

Options for this question:

1.After myopia surgery, the eyes can completely return to a non-nearsighted state

2.Myopia surgery cannot completely restore the eyes to a non-nearsighted state, it can only change the degree of myopia

3.Myopia surgery can not only change the degree of myopia but also eliminate fundus changes caused by myopia

4.Myopia surgery can only change the degree of myopia, but cannot eliminate fundus changes caused by myopia, so regular examinations are still needed

Q4_7. 【Single choice】Have you conducted continuous (regular) comprehensive eye health checks for your child?

Options for this question:

1.Yes

2.No

Q4_8. 【Single choice】Do you keep a continuous record of the results of your child's eye health checks?

Options for this question:

1.Yes, but often can't find the past records

2.Yes, all the records of the years are kept, and the eye condition of the child can be continuously tracked

3.No
